# Diabetes Self-Management and Health-Related Quality of Life among Primary Care Patients with Diabetes in Qatar: A Cross-Sectional Study

**DOI:** 10.3390/healthcare10112124

**Published:** 2022-10-25

**Authors:** Diana Alsayed Hassan, Fatema Helaluddin, Ozra Hajebi Chahestani, Omnia Mohamed, Nazmul Islam

**Affiliations:** 1 Department of Public Health, College of Health Sciences, QU Health, Qatar University, Doha 2713, Qatar; 2 Department of Health Research Methods, Evidence and Impact, McMaster University, Hamilton, ON L8S 4K1, Canada

**Keywords:** diabetes, self-management, health-related quality of life

## Abstract

Diabetes self-management (DSM) practices are an important determinant of health-related outcomes, including health-related quality of life (HRQOL). The purpose of this study is to explore DSM practices and their relationship with the HRQOL of patients with type 2 diabetes in primary health care centers (PHCCs) in Qatar. In this cross-sectional study, data were collected from PHCC patients with diabetes via interview-administered questionnaires by utilizing two instruments: the DSM questionnaire (DSMQ) and the HRQOL Short Form (SF-12). Frequencies were calculated for categorical variables and medians were calculated for continuous variables that were not normally distributed. A statistical comparison between groups was conducted using chi-square for categorical data. Binary logistic regression was utilized to examine the relationship between the significant independent factors and the dependent variables. A total of 105 patients completed the questionnaire, 51.4% of whom were male. Approximately half of the participants (48.6%) reported poor overall DSM practices, and 50.5% reported poor physical health quality of life (PC) and mental health quality of life (MC). Female participants showed significantly higher odds of reporting poor DSM than male participants (OR, 4.77; 95% CI, 1.92–11.86; *p* = 0.001). Participants with a secondary education (OR, 0.18; 95% CI, 0.04–0.81; *p* = 0.025) and university education (OR, 0.18; 95% CI, 0.04–0.84; *p* = 0.029) showed significantly lower odds of reporting poor DSM than participants with no/primary education. Older participants showed higher odds of reporting poor PC than younger participants (OR 11.04, 95% CI, 1.47–82.76 and OR 8.32; 95% CI, 1.10–62.86, respectively). Females also had higher odds for poor PC than males (OR 7.08; 95% CI, 2.21–22.67), while participants with a secondary (OR, 0.13; 95% CI, 0.03–0.62; *p* = 0.010) and university education (OR, 0.11; 95% CI, 0.02–0.57; *p* = 0.008) showed significantly lower odds of reporting poor MC. In conclusion, patients with diabetes reported poor overall DSM practices and poor HRQOL. Our findings suggest intensifying efforts to deliver culturally appropriate DSM education to patients and to empower patients to take charge of their health.

## 1. Introduction

Diabetes mellitus continues to be one of the most debilitating diseases. According to the International Diabetes Federation, the prevalence of diabetes in the Middle East and North African region is 12.8%, the highest in the world (IDF regions) [1]. Alarmingly, in Qatar, the prevalence of diabetes among adults is 15.5%, which is higher than the global prevalence [2]. It is projected that the prevalence of type 2 diabetes in Qatar will increase to at least 24% by 2050 [3].

It is well-documented in the literature that individuals with diabetes report a deteriorated quality of life compared to individuals with no chronic conditions. This could be due to certain clinical characteristics of diabetes, such as the required comprehensive daily-care activities; the presence of comorbidities; and diabetes complications, which is the single most important determinant of how persons with diabetes perceive their physical and mental quality of life [4,5].

Heath-related quality of life (HRQOL) is a multidimensional concept that has been defined as the subjective evaluation of an individual’s physical and mental health from the individual’s perspective [6]. An improved or sustained quality of life in general is the primary goal outcome of diabetes early diagnosis, self-management, and treatment [7,8]. In recent years, there has been an increased interest in evaluating the quality of life of individuals with diabetes and other chronic conditions. While diabetes, due to its demanding and progressive nature, can impair an individual’s perception of their quality of life, a deteriorated perception of quality of life can lead to poor self-care activities related to diabetes self-management [7].

The term self-management refers to the day-to-day activities that individuals undertake to minimize the negative outcomes and to prevent further complications of their chronic condition over the course of their illness [9]. Self-management behaviors for participants with diabetes mellitus represent a collection of actions that include undergoing treatment, self-monitoring glucose, exercising regularly, and managing diet, in addition to problem solving and reducing risks [10]. It is well-established in the literature that participants who are actively engaged in diabetes self-management have improved health outcomes [11,12].

Additionally, a magnitude of studies have pointed to the importance of DSM practices as a way to improve quality of life, making HRQOL a significantly important outcome for individuals with diabetes [13,14]. However, the relationship between diabetes self-management and quality of life among people with diabetes has not been explored in Qatar. The purpose of this study was to investigate DSM practices among participants with type 2 diabetes attending primary health care centers (PHCCs) in Qatar and to explore the relationship between participants’ DSM practices and their perceived quality of life.

## 2. Materials and Methods

### 2.1. Study Design

This is a multicenter, cross-sectional study conducted among participants with type 2 diabetes mellitus from seven primary health care centers in Qatar that offered clinical services for individuals with diabetes. Since the PHCC locations that offer diabetes clinical services are limited, we were bound by PHCC recommendations as to which clinics to approach.

### 2.2. Data Collection and Instruments

Data were collected utilizing interview-administered questionnaires from a convenience sample in patient waiting areas at the seven diabetes clinics. Three of the researchers interviewed patients in the waiting area after asking for their consent to participate in the study. The inclusion criteria included adult participants over the age of 18 with a type 2 diabetes diagnosis, who were fluent in English or Arabic, and who consented to participate in the study.

Our questionnaire included three sections:

1. Socio-demographic and clinical characteristics: Data on gender (male, female), education level (no formal education, primary education, secondary education, university education), age (4 categories), nationality (Qatari, non-Qatari), diabetes duration (less than 1 year, 1 year, 2 years, more than 2 years), number of comorbidities (none, 1, 2, more than 2 comorbidities), and HbA1c level were collected.
2. The Diabetes Self-Management Questionnaire (DSMQ) was utilized to assess patient’s self-management activities related to their diabetes. The tool is composed of 16 items, which are scored on a 4-point scale from 0 (does not apply to me) to 3 (applies to me very much). The DSMQ has 4 sub-scales, namely, Glucose Management (items 1, 4, 6, 10, 12), Dietary Control (items 2, 5, 9, 13), Physical Activity (items 8, 11, 15), and Health-Care Use (items 3, 7, 14). From the 16 items, 9 items that are worded negatively require reverse scoring. Item 16 requests an “overall rating” of self-care, and it is included in the sum scale. The total possible score can range between 0 and 48, with a higher score indicating more effective self-management behavior. Scale scores were calculated as sums of item scores and then transformed to a scale ranging from 0 to 10 for ease of interpretation [15]. The DSMQ is a reliable and valid tool that provides a measurement of diabetes self-management behaviors in relation to glycemic control [15,16]. The DSMQ was translated to Arabic using the standardized forward and backward translation method in order to validate the quality of the translated tool, and it was culturally adapted to the local context. Additionally, the Arabic versions of the questionnaires were reviewed by 10 bilingual adults and by two experts in diabetes.
3. The 12-item Health-Related Quality of Life Questionnaire Short Form (SF-12) was used to evaluate HRQOL. The SF-12 tool assesses patient’s perceived health status and constitutes two components: a physical health component (PC) and a mental health component (MC) [17,18]. Each parameter was calculated by applying an algorithm to generate a score from 0 to 100, with a higher score reflecting a better quality of life. We utilized the Arabic version of the tool, which has been determined to be a valid and reliable tool in previous studies [19,20,21].

Data collection occurred between 14 April and 24 April 2019.

### 2.3. Data Analysis

Data were analyzed using the Statistical Package for the Social Sciences (SPSS, version 24; IBM, Armonk, NY, USA). For a descriptive analysis, frequencies were calculated for categorical variables, and medians were calculated for continuous variables that were not normally distributed. A statistical comparison between groups was conducted using chi-square for categorical data. Binary logistic regression was utilized to examine the relationship between the significant independent factors and the dependent variables. The alpha level was set at 5% for statistical significance.

The PC, MC, and DSMQ variables were each further split into two groups based on the median scores. Participants who scored below the median were classified as “poor” and were given a score of “0”, while those who scored more than or equal to the median were categorized as “good” and were given a score of “1”.

### 2.4. Ethical Consideration

This study was approved by the Primary Health Care Centers Research Committee on 31 March 2019 (PHCC/RC/19/02/004) and the Qatar University Research Board on 14 April 2019 (QU-IRB 1054-EA/19). Informed consent was obtained from all participants involved in the study.

## 3. Results

The study included a total of 105 participants, 51.4% of whom were male. The majority of the participants had either secondary (41.9%) or a university education (40.0%). A total of 66.7% reported having diabetes for more than two years. A total of 47.6% of the participants reported having at least one comorbidity. Almost half of the participants reported having poor DSM (48.6%), poor PC (50.5%), and poor MC (50.5%). The mean HbA1c level was 7.6 ± 1.9 and was reported by less than half of the participants (45.7%) (Table 1).

The associations between the baseline characteristics of the participants with diabetes and their DSM status are presented in Table 2. The majority of females reported poor DSM (69.0% versus 30.0%; *p* < 0.001). There were no significant associations between age categories, nationality, diabetes duration, comorbidities, and HbA1C level with DSM status.

A further analysis of the DSM subscales releveled that participants with a poor physical activity status were more likely to be males (*p* = 0.001) and had diabetes for over 2 years (*p* = 0.024). Similarly, participants with diabetes for over 2 years had poor health care use compared to those with a shorter diabetes duration (79.0% versus 59.0%; *p* = 0.035). Additionally, females reported poorer glucose management than males (61% versus 39%, *p* = 0.048), as observed in Table 3.

The associations between the participant’s baseline characteristics and PC and MC QOL statuses are presented in Table 4. Females were more likely to report poor PC compared with males (*p* < 0.001). Participants with good DSM reported good PC (*p* < 0.001). The median HbA1C level was higher for participants with poor MC than for participants with good MC (*p* = 0.011). No association was found between DSM and HRQOL components.

The multivariable logistic regression analysis of significant factors associated with DSM among participants are presented in Table 5. Female participants showed a significantly higher odds of reporting poor DSM than male participants (OR, 4.77; 95% CI, 1.92–11.86; *p* = 0.001). Participants with secondary education (OR, 0.18; 95% CI, 0.04–0.81; *p* = 0.025) and university education (OR, 0.18; 95% CI, 0.04–0.84; *p* = 0.029) showed a significantly lower odds of reporting poor DSM than participants with no/primary education.

The multivariable logistic regression analysis of the significant factors associated with PC and MC of HRQOL among participants is presented in Table 6. Older participants (in the age categories of 42–50 and >50) showed higher odds of poor PC than younger participants (18–33 years of age) (OR 11.04, 95% CI, 1.47–82.76 and OR 8.32; 95% CI, 1.10–62.86, respectively). Females also had higher odds for poor PC than males (OR 7.08; 95% CI, 2.21–22.67). Participants with a secondary education (OR, 0.13; 95% CI, 0.03–0.62; *p* = 0.010) and university education (OR, 0.11; 95% CI, 0.02–0.57; *p* = 0.008) showed significantly lower odds of reporting poor MC than participants with no/primary education.

## 4. Discussion

This was a cross-sectional study that revealed important findings related to diabetes self-management practices, health-related quality of life, and associated factors among a sample of patients with diabetes of the primary health care system in Qatar.

Our study found that approximately half of the participants reported poor overall DSM practices, as revealed by the DSM median score. This result is comparable to that of similar studies conducted in Kuwait and Saudi Arabia, which reported similar DSM median scores of 6.5 and 5.04, respectively, indicating poor self-care habits among their participants [22,23]. Barriers to DSM have been reported extensively in the literature. Locally, in Qatar, a qualitative study shed light on the barriers reported by 29 participants with diabetes [24]. The authors found similar barriers reported in other studies, such as work-related stress, the cost of testing strips, and long working hours; all external barriers. However, with Qatar being a country that hosts 94 other nationalities, culture also plays a major role in how individuals with diabetes perceive their DSM [24].

Furthermore, a related striking result was that less than half of our participants knew their most recent HbA1c value. These findings reflect the low knowledge levels of our participants. A similar study that surveyed adults with type 2 diabetes on their knowledge, attitudes, and practices (KAP) in Qatar found that participants reported poor knowledge related to diabetes [25]. Another KAP study also from Qatar that surveyed 2400 people from the general public found that the knowledge component had the lowest score. As a matter of fact, 69% of their participants scored low on knowledge related to normal fasting glucose levels [26]. Other studies from the region have confirmed low levels of knowledge related to diabetes [27,28]. Despite the extensive evidence that diabetes self-management education (DSME) is an effective and available resource in improving knowledge related to diabetes, DSM activities, and quality of life, in addition to other diabetes-related clinical outcomes [11,12], attendance remains poor, and, therefore, DSME remains an underutilized resource [29,30].

Education level was found to be a significant predictor in reporting better DSM and better MC in our study. Findings from other studies examining the relationship between education and DSM have been inconsistent. A similar study from the region in which participants with diabetes were surveyed in a primary health care setting about DSM found that participants with formal education reported better DSM [31]. On the contrary, a systematic review found that education was not a significant factor in reporting better DSM [32]. Education level or attainment, a long-standing indicator of socioeconomic status, is a complex and multidimensional factor that should not be measured the traditional way. A limitation to our study is that participants’ previous knowledge and training related to DSM were not taken into account, which may have influenced our results [33].

Furthermore, our study revealed important gender differences in diabetes self-management, as females reported worse DSM habits than males overall, which remained significant in the regression analysis. When we further analyzed the DSMQ, we found that females also reported poorer glucose management than males. Glucose management is an essential cornerstone of DSM that, if not managed properly, could lead to further complications. Sex differences in diabetes self-management and disease outcomes exist, as seen in other studies. Sex differences have been attributed to biological factors, such as differences in hormonal pathophysiology [34,35]. Gender differences, however, are related to complex psychosocial processes that shape human behavior and in turn manipulate the clinical outcomes of diabetes [34]. For example, women in general have poorer glycemic control and are less likely to reach their A1c targets [36,37], which our study results are in line with. Considering the local psychosocial and cultural contexts when comparing our results to other studies conducted in the Gulf Cooperation Council (GCC) region, we found that our study results are in line with another study from Kuwait in which they found that men scored significantly better on the DSMQ than women [22].

Gender differences in DSM are clearly reported in the literature. Females tend to assume a responsible caregiving role in diabetes self-management toward family members, especially toward their spouses. The literature shows that the support females with diabetes receive from family members, especially from spouses, is less compared to the support that males receive from their female spouses [38,39]. This might be linked to our finding that women reported poorer PC than men. In most studies that examined gender differences in quality of life among individuals with diabetes, it was found that women tended to report worse outcomes than men [5,40]. A study in Qatar that examined the quality of life predictors of individuals with diabetes also found that females tend to report a lower quality of life than males [41].

In addition to gender, we found that older participants reported poorer PC than younger participants. The link between diabetes and the impairment of HRQOL has long been studied and documented. A longitudinal study that included data from 26,344 participants found that participants with a type 2 diabetes M diagnosis had a fivefold increase in the odds of reporting a significantly poorer quality of life [42]. Another longitudinal study that assessed patients with diabetes at a five-year follow-up point also found a deterioration in patients’ reported HRQOL over time [43].

Although our study did not find a direct link at the multivariate level between DSM and HRQOL, it sheds light on other factors associated with DSM and HRQOL. This study had limitations. A main limitation is that it is a cross-sectional study, in which causal relationships cannot be established, in addition to the small sample size of patients, making our results not generalizable.

## 5. Conclusions

Our study results highlight the importance of providing patients with diabetes with diabetes self-management education to enhance patients’ health literacy, knowledge, and skills needed to successfully self-manage diabetes and to prevent its complications, and ultimately to improve quality of life. Furthermore, these programs should be culturally adapted to suit local needs and to address gender gaps.

## Figures and Tables

**Table 1:** Baseline characteristics of the patients.

Variables	Frequency	Percentage
Age categories		
18–33 years	16	15.2
34–41 years	22	21.0
42–50 years	31	29.5
>50 years	36	34.3
Gender		
Male	54	51.4
Female	51	48.6
Education level		
No/primary education	19	18.1
Secondary education	44	41.9
University education	42	40.0
Duration of Diabetes		
≤2 years	35	33.3
>2 years	70	66.7
Comorbidities		
None	36	34.3
1	50	47.6
2	11	10.5
>2	8	7.6
DSM		
Poor	51	48.6
Good	54	51.4
PC HRQOL		
Poor	53	50.5
Good	52	49.5
MC HRQOL		
Poor	53	50.5
Good	52	49.5
**Variables**	**Mean (±SD)**	**Median (IQR)**
HbA1C (*n* = 48)	7.6 (±1.9)	7.1 (6.3–8.4)
DSM score	7.2 (±1.8)	7.1 (5.8–8.8)
PC HRQOL score	47.8 (±9.8)	48.2 (40.7–56.6)
MC HRQOL score	46.7 (±10.3)	47.1 (38.9–55.6)

Continuous variables presented as mean ± standard deviations. Categorical variables presented as *n* (%).

**Table 2:** Diabetes self-management status according to baseline characteristics of participants.

Diabetes Self-Management			
Variables	Poor (*N* = 51) *n* (%)	Good (*N* = 54) *n* (%)	*p*-Value
Age categories			
18–33 years	9 (18.0%)	7 (13.0%)	0.770
34–41 years	11 (22.0%)	11 (20.0%)	
42–50 years	16 (31.0%)	15 (28.0%)	
>50 years	15 (29.0%)	21 (39.0%)	
Gender			
Male	16 (31.0%)	38 (70.0%)	<0.001
Female	35 (69.0%)	16 (30.0%)	
Education level			
No/primary education	14 (27.0%)	5 (9.0%)	0.052
Secondary education	19 (37.0%)	25 (46.0%)	
University education	18 (35.0%)	24 (44.0%)	
Duration of Diabetes			
≤2 years	19 (37.0%)	16 (30.0%)	0.420
>2 years	32 (63.0%)	38 (70.0%)	
Comorbidities			
None	19 (37.0%)	17 (31.0%)	0.830
1	23 (45.0%)	27 (50.0%)	
2	6 (12.0%)	5 (9.0%)	
>2	3 (6.0%)	5 (9.0%)	
HbA1C [Median(IQR)]	8.0 (6.4–8.4)	7.0 (6.2–8.3)	0.256

**Table 3:** Diabetes self-management subscales status according to participants’ baseline characteristics.

	Glucose Management			Dietary Control			Physical Activity			Health Care Use		
Variables	Poor (*N* = 41) *n* (%)	Good (*N* = 64) *n* (%)	*p*-Value	Poor (*N* = 22) *n* (%)	Good (*N* = 83) *n* (%)	*p*-Value	Poor (*N* = 30) *n* (%)	Good (*N* = 75) *n* (%)	*p*-Value	Poor (*N* = 39) *n* (%)	Good (*N* = 66) *n* (%)	*p*-Value
Age												
18–33 years	5 (12.0%)	11 (17.0%)	0.560	3 (14.0%)	13 (16.0%)	0.760	3 (10.0%)	13 (17.0%)	0.096	5 (13.0%)	11 (17.0%)	0.120
34–41 years	11 (27.0%)	11 (17.0%)		3 (14.0%)	19 (23.0%)		5 (17.0%)	17 (23.0%)		7 (18.0%)	15 (23.0%)	
42–50 years	13 (32.0%)	18 (28.0%)		8 (36.0%)	23 (28.0%)		6 (20.0%)	25 (33.0%)		8 (21.0%)	23 (35.0%)	
>50 years	12 (29.0%)	24 (38.0%)		8 (36.0%)	28 (34.0%)		16 (53.0%)	20 (27.0%)		19 (49.0%)	17 (26.0%)	
Gender												
Male	16 (39.0%)	38 (59.0%)	0.048	10 (45.0%)	44 (53.0%)	0.630	23 (77.0%)	31 (41.0%)	0.001	24 (62.0%)	30 (45.0%)	0.160
Female	25 (61.0%)	26 (41.0%)		12 (55.0%)	39 (47.0%)		7 (23.0%)	44 (59.0%)		15 (38.0%)	36 (55.0%)	
Education level												
No/primary education	6 (15.0%)	13 (20.0%)	0.810	4 (18.0%)	15 (18.0%)	0.950	5 (17.0%)	14 (19.0%)	0.580	5 (13.0%)	14 (21.0%)	0.580
Secondary education	18 (44.0%)	26 (41.0%)		10 (45.0%)	34 (41.0%)		15 (50.0%)	29 (39.0%)		17 (44.0%)	27 (41.0%)	
University education	17 (41.0%)	25 (39.0%)		8 (36.0%)	34 (41.0%)		10 (33.0%)	32 (43.0%)		17 (44.0%)	25 (38.0%)	
Duration of Diabetes												
≤2 years	13 (32.0%)	22 (34.0%)	0.830	7 (32.0%)	28 (34.0%)	1.00	5 (17.0%)	30 (40.0%)	0.024	8 (21.0%)	27 (41.0%)	0.035
>2 years	28 (68.0%)	42 (66.0%)		15 (68.0%)	55 (66.0%)		25 (83.0%)	45 (60.0%)		31 (79.0%)	39 (59.0%)	
Comorbidities												
None	14 (34.0%)	22 (34.0%)	0.970	9 (41.0%)	27 (33.0%)	0.840	11 (37.0%)	25 (33.0%)	0.500	13 (33.0%)	23 (35.0%)	0.530
1	19 (46.0%)	31 (48.0%)		9 (41.0%)	41 (49.0%)		15 (50.0%)	35 (47.0%)		17 (44.0%)	33 (50.0%)	
2	5 (12.0%)	6 (9.0%)		2 (9.0%)	9 (11.0%)		1 (3.0%)	10 (13.0%)		4 (10.0%)	7 (11.0%)	
>2	3 (7.0%)	5 (8.0%)		2 (9.0%)	6 (7.0%)		3 (10.0%)	5 (7.0%)		5 (13.0%)	3 (5.0%)	
HbA1C [Median(IQR)]	7.0 (6.4–8.0)	7.4 (6.2–8.6)	0.655	7.1 (6.4–8.2)	7.1 (6.1–8.7)	0.892	7.0 (6.4–8.3)	7.4 (6.2–8.4)	0.779	7.0 (6.4–8.2)	7.7 (6.2–8.5)	0.620

**Table 4:** PC and MC of HRQOL status according to baseline characteristics of participants with diabetes attending PHCC in Qatar.

	PC HRQOL Status					
Variables	Poor (*N* = 53) *n* (%)	Good (*N* = 52) *n* (%)	*p*-Value	Poor (*N* = 53) *n* (%)	Good (N = 52) *n* (%)	*p*-Value
Age categories						
18–33 years	6 (11.0%)	10 (19.0%)	0.273	8 (15.0%)	8 (15.0%)	0.296
34–41 years	10 (19.0%)	12 (23.0%)		14 (26.0%)	8 (15.0%)	
42–50 years	20 (38.0%)	11 (21.0%)		17 (32.0%)	14 (27.0%)	
>50 years	17 (32.0%)	19 (37.0%)		14 (26.0%)	22 (42.0%)	
Gender						
Male	17 (32.0%)	37 (71.0%)	<0.001	27 (51.0%)	27 (52.0%)	0.92
Female	36 (68.0%)	15 (29.0%)		26 (49.0%)	25 (48.0%)	
Education level						
No/primary education	13 (25.0%)	6 (12.0%)	0.180	12 (23.0%)	7 (13.0%)	0.434
Secondary education	22 (42.0%)	22 (42.0%)		20 (38.0%)	24 (46.0%)	
University education	18 (34.0%)	24 (46.0%)		21 (40.0%)	21 (40.0%)	
Duration of Diabetes						
≤2 years	19 (36.0%)	16 (31.0%)	0.581	19 (36.0%)	16 (31.0%)	0.581
>2 years	34 (64.0%)	36 (69.0%)		34 (64.0%)	36 (69.0%)	
Comorbidities						
None	20 (38.0%)	16 (31.0%)	0.387	16 (30.0%)	20 (38.0%)	0.105
1	21 (40.0%)	29 (56.0%)		31 (58.0%)	19 (37.0%)	
2	7 (13.0%)	4 (8.0%)		4 (8.0%)	7 (13.0%)	
>2	5 (9.0%)	3 (6.0%)		2 (4.0%)	6 (12.0%)	
DSM						
Poor	35 (66.0%)	16 (31.0%)	<0.001	27 (51.0%)	24 (46.0%)	0.623
Good	18 (34.0%)	36 (69.0%)		26 (49.0%)	28 (54.0%)	
HbA1C [Median(IQR)]	8.1(6.5–8.7)	7(6.2–8.0)	0.062	8.3 (7.6–8.8)	6.8 (6.2–7.4)	0.011

PC HRQOL, physical health component of health-related quality of life; MC HRQOL, mental health component of health-related quality of life; DSM, diabetes self- management.

**Table 5:** Multivariable logistic regression analysis of diabetes self-management among diabetic participants attending PHCC in Qatar.

Variables	Crude OR	*p*-Value	95% CI	Adjusted OR	*p*-Value	95% CI
Overall Diabetes Self-Management						
Gender						
Male	Reference			Reference		
Female	5.20	<0.001	2.26–11.93	4.77	0.001	1.92–11.86
Education level						
No/primary education	Reference			Reference		
Secondary education	0.27	0.031	0.08–0.89	0.18	0.025	0.04–0.81
University education	0.27	0.030	0.08–0.89	0.18	0.029	0.04–0.84
Subscales						
Glucose Management						
Gender						
Male	Reference			Reference		
Female	2.28	0.043	1.02–5.09	2.42	0.050	0.99–5.87

**Table 6:** Multivariable logistic regression analysis of poor PC and MC of QOL among participants with diabetes attending PHCC in Qatar.

QOL	Crude OR	*p*-Value	95% CI	Adjusted OR	*p*-Value	95% CI
PC HRQOL						
Age categories						
18–33 years	Reference			Reference		
34–41 years	1.39	0.624	0.37–5.17	2.44	0.314	0.43–13.87
42–50 years	3.03	0.082	0.87–10.59	11.04	0.019	1.47–82.76
>50 years	1.49	0.516	0.45–4.98	8.32	0.040	1.10–62.86
Gender						
Male	Reference			Reference		
Female	5.22	<0.001	2.27–12.1	7.08	0.001	2.21–22.67
MC HRQOL						
Education level						
No/primary education	Reference			Reference		
Secondary education	0.49	0.201	0.16–1.47	0.13	0.010	0.03–0.62
University education	0.58	0.342	0.19–1.77	0.11	0.008	0.02–0.57

PC HRQOL, physical health component of health-related quality of life; MC HRQOL, mental health component of health-related quality of life.

## Data Availability

The data presented in this study are available on request from the corresponding author.
